# Contrasting Epidemic Histories Reveal Pathogen-Mediated Balancing Selection on Class II MHC Diversity in a Wild Songbird

**DOI:** 10.1371/journal.pone.0030222

**Published:** 2012-01-23

**Authors:** Dana M. Hawley, Robert C. Fleischer

**Affiliations:** Center for Conservation and Evolutionary Genetics, Smithsonian Institution, Washington D.C., United States of America; Michigan State University, United States of America

## Abstract

The extent to which pathogens maintain the extraordinary polymorphism at vertebrate Major Histocompatibility Complex (MHC) genes via balancing selection has intrigued evolutionary biologists for over half a century, but direct tests remain challenging. Here we examine whether a well-characterized epidemic of Mycoplasmal conjunctivitis resulted in balancing selection on class II MHC in a wild songbird host, the house finch (*Carpodacus mexicanus*). First, we confirmed the potential for pathogen-mediated balancing selection by experimentally demonstrating that house finches with intermediate to high multi-locus MHC diversity are more resistant to challenge with *Mycoplasma gallisepticum*. Second, we documented sequence and diversity-based signatures of pathogen-mediated balancing selection at class II MHC in exposed host populations that were absent in unexposed, control populations across an equivalent time period. Multi-locus MHC diversity significantly increased in exposed host populations following the epidemic despite initial compromised diversity levels from a recent introduction bottleneck in the exposed host range. We did not observe equivalent changes in allelic diversity or heterozygosity across eight neutral microsatellite loci, suggesting that the observations reflect selection rather than neutral demographic processes. Our results indicate that a virulent pathogen can exert sufficient balancing selection on class II MHC to rescue compromised levels of genetic variation for host resistance in a recently bottlenecked population. These results provide evidence for Haldane's long-standing hypothesis that pathogens directly contribute to the maintenance of the tremendous levels of genetic variation detected in natural populations of vertebrates.

## Introduction

A potential role for pathogens in maintaining the extraordinary polymorphism at the vertebrate Major Histocompatibility Complex (MHC) was suspected long before the molecular role of MHC genes was understood [1]. Today, the critical function of MHC glycoproteins in antigen recognition and presentation, a hallmark of vertebrate immune specificity, is well understood [2]. In contrast, our understanding of the evolutionary processes maintaining the high polymorphism at MHC genes remains primarily indirect. Assortative mate choice [e.g. 3], pathogen-mediated balancing selection [e.g. 4]–[6], and spatial variation in parasite-mediated selection pressures [e.g. 7]–[9] have all been proposed as potential mechanisms maintaining MHC polymorphism in wild populations. Several lines of evidence provide indirect support for the importance of pathogen-mediated balancing selection, which can act via heterozygote advantage or negative frequency-dependent selection: first, heterozygote advantage at MHC genes has been documented for a suite of vertebrate systems [10]–[12]; second, nucleotide substitution patterns at MHC exons exhibit molecular signatures of balancing selection [e.g. 13]; and third, transpecific polymorphisms in several vertebrate groups document the maintenance of MHC variants over extraordinarily long evolutionary time scales [e.g. 14]. Opportunities to directly test whether pathogens have exerted selection on non-model and non-human MHC genes have been extremely rare [15]–[17].

Avian systems are particularly promising yet challenging candidates for the study of the evolutionary ecology of MHC genes [18]. Because of the large distinctions between the structure of mammalian and chicken MHC, which is considered “minimally essential”, domestic chickens were among the first vertebrates to reveal a strong role for MHC genetics in pathogen resistance [19]. However, significant structural differences exist between the “minimally essential” chicken MHC and the larger passerine MHC gene complex: passerine birds show high levels of duplication across class II MHC loci, with up to 20 loci detected in the common yellowthroat, *Geothlypis trichas* [20], and variable numbers of loci detected for other passerine species examined [21]–[22]. Nonetheless, associations between MHC variants and avian malaria susceptibility have been detected in both passerine systems examined to date: house sparrows [23]–[24] and great reed warblers [25].

The large extent of gene duplication which characterizes passerine MHC structure necessitates a multi-locus approach to characterize functionally similar loci of unknown homology [18]. This pattern is not limited to birds: variation in MHC class II copy number and/or allele sharing across loci has been documented for a suite of vertebrate systems [e.g. 26]–[29] and likely represents a conserved vertebrate strategy for generating functional class II MHC diversity within and among loci. Although class II MHC alleles have been shown to covary with parasite load in a variety of natural systems [e.g. 30]–[32], the fitness benefits of variant diversity are less well documented [33]–[34]. Here we use a common North American passerine species, the house finch (*Carpodacus mexicanus*), to examine whether a well-characterized pathogen epidemic caused by *Mycoplasma gallisepticum* (hereafter “MG”) altered levels of individual variant diversity or molecular signatures of selection at the region of class II MHC containing the peptide-binding region. We chose to examine class II MHC due to the documented importance of this MHC subtype in mediating responses to bacterial infections by initiating T-cell responses to extracellular antigens [2].

The host jump of MG from commercial poultry into free-ranging house finches in eastern North America occurred in the early 1990s, and has been unusually well documented due to the involvement of “citizen scientist” birdwatchers who can readily distinguish the conjunctival signs associated with MG infection in house finches [35]. Initial Mycoplasmal conjunctivitis epidemics resulted in up to 60% declines in house finch population sizes [36] and a rapid spatial spread of MG infection within eastern, North American populations [35]. Although MG has recently been documented in the western, native North American range of house finches [37], these native populations were spatially isolated from the source of disease introduction and therefore unaffected by the MG epidemic from 1994–2004, when eastern populations were undergoing strong selection from annual epidemics [36]. These contrasting epidemic histories of native and introduced house finch populations offer a natural geographic test of pathogen-mediated balancing selection on host MHC. Furthermore, these populations have distinct demographic histories that began well prior to MG emergence, but render the system particularly interesting for examining how host MHC diversity both influences and responds to a pathogen epidemic: eastern house finch populations were derived from a small introduction of native birds in the 1940s [38], and both mtDNA and microsatellite genetic signatures of a recent bottleneck are present in eastern populations [39]–[40]. However, no study to date has examined whether the introduction of eastern house finches compromised diversity at MHC loci, a pattern that may have facilitated the emergence and rapid spread of the MG epidemic through introduced populations. Our study takes advantage of contrasting geographic differences in both host genetics and MG dynamics in native versus introduced house finches to test for signatures of pathogen-mediated balancing selection on class II MHC present only in exposed populations.

We used paired temporal comparisons to examine changes in MHC diversity across equivalent time periods (∼10 years) for exposed, eastern North American house finches versus unexposed, western populations (final samples for both populations were collected by 2004, prior to MG establishing itself in the western range of the host). We characterized individual-level MHC diversity as the number of segregating variants with unknown locus homology, and we amplified and sequenced a representative subset of variants to obtain information on sequence identity and variant frequency. Our diversity and sequence measures were based on a portion of exon 2 of the class II MHC in house finches, a region well characterized in several passerine species [41] due to its inclusion of the putative peptide-binding region (PBR). Since the PBR must precisely match a foreign antigen in order to effectively initiate a T-cell response, this region contains the vast majority of genetic polymorphism found throughout the exon. As a control gene marker not predicted to be under selection by MG, we also amplified eight nuclear microsatellite loci for our paired comparisons of pre- and post-epidemic in exposed versus unexposed populations. We predicted that exposed host populations would show signatures of balancing selection on MHC sequences and individual-level variant diversity, but not at putatively neutral microsatellite loci following the MG epidemic. We also test whether there is evolutionary potential for pathogen-mediated balancing selection by examining the relationship between individual-level MHC diversity and disease response for a suite of experimentally infected captive finches.

## Materials and Methods

### (a) Ethics statement

All procedures involving animals were approved by Cornell University's Institutional Animal Care and Use Committee (Protocol 00-90) and were carried out in strict accordance with the standards published in the National Institute of Health's *Guide for the Care and Use of Laboratory Animals*.

### (b) Museum sampling

We used a suite of house finch tissues, museum specimens, and blood samples in order to test for signatures of balancing selection at house finch class II MHC. Tissue and toe pad samples of house finches collected across an 18 year period (1971 to 1993) were obtained from the Louisiana Museum of Natural History, the Smithsonian Museum of Natural History, and the Cornell University Museum of Vertebrates (Table 1). In order to confirm that our museum extractions were robust, we obtained 6 toe pad samples from the identical individuals for which we had tissue samples from the Louisiana State University Museum of Natural Science: all multi-locus amplifications produced identical numbers of segregating variants via single-strand conformation polymorphism (SSCP). As a second test, we compared the average amplification success for tissues versus toe-pads in our *pre-epidemic* eastern sample only: the average SSCP variants obtained per individual from toe-pad samples (2.81 bands) and tissue samples (2.75 bands) were statistically identical, indicating that allelic drop-out from toe-pad samples did not influence our results.

**Table 1:** Sampling information for all house finches included in the study.

No.	Pre-/Post	Pop.	Collection Dates	Location (All samples within USA)	Museum	Type
n = 15	Pre-	Eastern	1988–1993	Tompkins Co., NY	CMV	Toe pads
n = 3	Pre-	Eastern	1988–1989	Bucks Co., PA	CMV	Toe pads
n = 4	Pre-	Eastern	1990–1992	Acadia and Iberville Parish, LA	LSU	Tissue/Toe pads
n = 9	Pre-	Eastern	1984–1988	Fairfax & Loudon Co., Falls Church & Arlington Cities, VA	NMNH	Toe pads
n = 9	Pre-	Eastern	1981–1987	Charles, Montgomery, Talbot, Waldorf Co., MD	NMNH	Toe pads
**n = 40**	**Pre-MG East Total**					
n = 57	Post-	Eastern	2001–2004	Tompkins Co., New York	N/A	Blood
**n = 57**	**Post-MG East total**					
n = 2	Pre-	Western	1988–1990	San Bernadino Co., CA	LSU	Tissue/Toe pads
n = 2	Pre-	Western	1986–1993	Jefferson Davis & Atascosa Co., TX	LSU	Tissue/Toe pads
n = 3	Pre-	Western	1972–1974	Monterey Co., CA	NMNH	Toe pads
n = 1	Pre-	Western	1971	Grant Co., NM	NMNH	Toe pads
**n = 8**	**Pre-Control West Total**					
n = 15	Post-	Western	2004	Contra Costa Co., California, USA	N/A	Blood
**n = 15**	**Post-Control West Total**					

CMV = Cornell Museum of Vertebrates; NMNH = Smithsonian National Museum of Natural History, LSU = LSU Museum of Natural Science; No. = number; MG = *Mycoplasma gallisepticum*.

### (c) DNA extraction

DNA from blood and tissue samples was isolated using Qiagen Tissue Kits (Qiagen, Inc). Since a large proportion of our pre-epidemic DNA samples were obtained from museum toe-pads collected prior to MG emergence, all pre-PCR laboratory work was conducted using stringent ancient DNA methodologies in an isolated and dedicated facility as described previously [42]. We isolated museum-quality DNA using phenol-chloroform extraction and centrifugal dialysis. To control for potential contamination in our dedicated facility, we included a ratio of 1∶6 “blank” extractions for each round of DNA extraction, and a ratio of 1∶4 negative and “blank” extraction controls for each PCR. Our “blank” extractions and negative controls were never observed to amplify in a PCR reaction, indicating that our techniques prevented contamination at that scale. Finally, to control for the possibility of null alleles in our museum-quality DNA at the eight amplified microsatellite loci, we amplified all heterozygotes at least twice to verify results and all homozygotes up to seven independent times before concluding the absence of a second allele.

### (d) Class II MHC characterization

We used published primers *PBR 1.3* and *PBR 1.5* [15], designed for closely-related Hawaiian honeycreepers, to initially amplify the relevant MHC exon by polymerase chain reaction and separated fragments using SSCP to avoid the formation of heteroduplex products [43]. Once we obtained sufficient preliminary sequence, we designed species-specific primers *HofiEx2F* (5′ AGT GTT ACT ACA CCA ACG GCA) and *HofiExR4* (5′ GTT GTG CCG GCA GTA CCT) and used these to amplify 209-bp fragments, including primers, for the current study. Cycling conditions for the initial amplification began with 10 min at 94°C, followed by 40 cycles of 94°C for 50 sec, 51°C for 50 sec, and 72°C for 50 sec, and ended with a 10 min 72°C extension. PCR products re-amplified from extracted SSCP bands (see below) were subjected to similar cycling conditions but the annealing temperature was increased to 52°C and the number of cycles reduced to 30 in order to minimize potential contamination from nearby gel bands.

We used two multi-locus approaches —cloning and SSCP— to characterize functionally similar class II MHC sequences of unknown homology [18]. As a result, we use the term “variant” in place of “allele” to refer to all amplicons, which were universally of unknown locus identity.

#### Cloning

We directly cloned PCR products from a total of 16 individuals, including the two individuals for which we had both DNA and cDNA (see below). We ligated fresh PCR product into pCR® 4-TOPO® using a TOPO TA Cloning Kit (Invitrogen K4530-20) and transformed plasmids into One Shot® Mach1-T1 Chemically Competent *E. coli* (Invitrogen C8620-03). Transformed cells were grown on ampicillin-coated plates overnight, and 16 colonies were randomly selected per individual, amplified by PCR directly following colony growth using M13 primers and sequenced. Due to the high potential for mosaic, or artifactual sequences, as well as cloning errors, we only included sequences obtained from multiple colonies.

#### Single Stranded Conformational Polymorphism

We quantified individual-level MHC diversity as the number of segregating bands produced from a single multi-locus PCR reaction. To separate variants in each PCR that differed by conformation rather than size, we used a 0.5× MDE® gel (Lonza #50620, Rockland, ME) with 0.6× TBE, 20 uL Temed and 200 uL 10% ammonium persulphate. We denatured 10 uL unpurified PCR product and 10 uL loading buffer (.01 M NaOH, 0.18% Blue Dextran, and 0.8% formamide) for 10 min at 95°C and then snap froze the samples before electrophoresing them on an OWL Vertical System (Model ADJ-2) at 4°C and 200–300 W for 18–24 hours in 0.6× TBE running buffer. SSCP gels were stained using Gelstar and visualized on UV light. We extracted visualized bands from each individual using wide-bore 10 uL pipet tips, placing the gel core immediately into PCR master mix for amplification, purification (Qiaquick Purification Kits), and sequencing on an ABI 3100 (Applied Biosystems).

Due to the potential for ambiguity when scoring gels, all gels were scored blind to individual identity a total of three times. The median value was used for all further analyses. We confirmed the validity of our SSCP methods by running single variants isolated via cloning side-by-side our multi-locus amplifications of individuals known to harbor the cloned variant. In every case, banding patterns confirmed that the isolated cloned variant was present in the multi-locus amplification of the individual from which we had previously amplified that variant via SSCP. Furthermore, SSCP band segregation patterns and the sequence amplified from each band were repeatable within individuals on multiple gel runs. Because lighter sub-bands universally did not sequence cleanly following band extraction and reamplification, we did not include these potential PCR artifacts in our analysis of variant number.

#### cDNA comparison

We extracted mRNA from two house finch spleens stored at a 1∶10 ratio in RNA*later* (Qiagen) using Trizol® (Invitrogen). We synthesized cDNA from our extracted RNA using the AccuScript High Fidelity 1^st^-strand cDNA synthesis Kit (Strategene 200820). We amplified MHC from the cDNA using our primer set, cloned PCR product from cDNA and corresponding DNA for both individuals, and performed side-by-side SSCP separation. Variants obtained from both cloning and SSCP were identical for both house finch individuals, indicating that the amplified loci are expressed in house finch spleens.

In total, we obtained 210 unique sequences from 120 house finches comprising 55 DNA variants [HQ202977-HQ203031]. The number of segregating bands per individual as determined via SCCP, which served as our measure of individual MHC diversity, varied from 1 to 9, with a mean number of 3.51 variants per individual. Because we were unable to obtain sequence for all variants detected via SSCP, we use SSCP diversity (the number of segregating bands) as our measure of individual-level MHC diversity. We use the sequenced variants as representative subsets from each population for molecular analyses. Due to the large number of variants detected at very low frequency (Table 2), we were unable to conduct robust statistical tests on differences in variant frequency between our four populations.

**Table 2:** The frequencies of DNA variants of a fragment of exon 2 of the class II MHC detected in the sampled house finch populations.

Variant	Pre-MG	Post-MG	Pre-Control	Post-Control
1	0.05	0.053	0.125	0.267
2	0.175	0.193	0.25	0.0667
3	0.075	0.035	0.125	0
4	0.1	0.053	0	0
5	0.25	0.16	0.13	0.13
6	0.05	0.0175	0.125	0
7	0.15	0.053	0	0
8	0.025	0.0175	0	0
9	0	0.053	0	0
10	0.1	0.09	0	0.13
11	0.05	0.053	0	0
12	0	0.035	0.125	0.13
13	0.025	0.035	0	0
14	0.05	0.053	0	0
15	0.075	0.14	0.125	0
16	0.025	0	0.125	0
17	0.025	0.053	0	0
18	0	0.018	0.125	0.067
19	0.025	0.07	0	0
20	0	0.018	0	0.067
21	0.15	0.035	0	0
22	0.025	0.018	0	0
23	0	0.07	0	0
24	0.05	0.14	0	0
25	0.075	0	0	0
26	0	0.053	0	0
27	0.025	0.0175	0	0
28	0.025	0.0175	0	0.067
29	0.025	0.0175	0	0
30	0.05	0	0	0
31	0.025	0	0.125	0
32	0.025	0.053	0.125	0.067
33	0.03	0	0	0.13
34	0	0.018	0.125	0
35	0.025	0	0.125	0
No. singletons*/N	2/40	4/57	4/8	8/15

*Singleton variants were detected in only a single individual in the global sample.

N = no. individuals sampled in each population; MG = *Mycoplasma gallisepticum*.

### (e) Microsatellites genotyping

All microsatellite markers used had a null allele frequency less than 0.10 in our population. Two out of 32 population-locus combinations significantly deviated from the assumptions of Hardy-Weinberg equilibrium (α = 0.002; Hofi20 in the post-epidemic eastern population and Hofi52 in the pre-control western population). Two of twenty-eight locus pairs showed significant linkage disequilibrium (α = 0.002; Hofi3 and Hofi5; Hofi20 and Lox3). However, population-level comparisons revealed that LD between these locus pairs was only detected in one of four populations (pre-epidemic eastern), suggesting that the loci are unlikely to be tightly linked. Microsatellite diversity indices were analyzed using GENEPOP v. 3.4 [44] and FSTAT v2.9.3.2 [45].

### (f) Experimental MG challenge

In order to link MHC diversity with resistance to MG, we used 39 birds that have been subject to experimental disease challenges in previous studies [46, and A. and K. Dhondt, unpub. data]. In brief, individually housed finches were inoculated bilaterally in the palpebral conjunctiva with 0.05 mL (3.24×10^5^ CFUs) of a 1994 MG isolate from a VA house finch (7994-1 6P). Eye lesions were scored biweekly for 10 weeks following infection on a 0 to 3 scale: 1 for minor swelling, 2 for moderate swelling and eversion of conjunctival tissue, and 3 if the eye was nearly hidden by swelling and crusted exudate. Mortality following experimental infection was low (2.5%) relative to that experienced in the wild (up to 60%) during the early stages of the epidemic [36], likely due to the presence of *ad libitum* food and the absence of predators in captivity. We quantified disease severity for each individual as the sum of weekly conjunctival symptoms, and square-root transformed values in order to meet the assumptions of normality [47].

### (g) Statistical analyses

We used JMP 9.0 (SAS Institute, Cary, NC) for all non-molecular statistical analyses. For the experimental challenge data, we used mixed linear models to examine associations between individual-level variant diversity and disease severity across individuals in our experimental population. We included bird identity as a random effect and microsatellite heterozygosity, which has been shown to influence disease severity in house finches [47], as a covariate. Because prior studies have found that intermediate levels of MHC diversity are associated with the highest pathogen resistance [29], [48], we included both first and second-order relationships between individual-level MHC diversity and MG resistance in our model. We used a log-likelihood ratio test evaluated against a Chi-square distribution to determine whether including a second-order relationship for MHC diversity improved model fit.

To examine temporal changes in individual-level MHC diversity and microsatellite diversity (allelic diversity and heterozygosity) prior to and following the epidemic, we used general linear models, including population (western versus eastern), time (pre- versus post-epidemic), and their interaction in our model. For the microsatellite analyses, we considered locus-specific metrics of allelic diversity and heterozygosity for each population as independent data points in our model.

We defined rare MHC variants as those that were only present in a single individual in each population (e.g. pre-epidemic east, post-epidemic east, pre-control west, post-control west). To compare the proportion of rare MHC variants in each population, we standardized for the differences in sample size among populations (Table 1) by repeatedly subsampling our eastern population data sets, which were significantly larger. We used a random number generator to subsample without replacement, generating a sample size of n = 8 for the pre-epidemic eastern population and a sample size of n = 14 for our post-epidemic eastern population. The number of rare variants in each of 10 eastern subsamples were averaged and compared to the true values generated from the equivalently sized western populations.

We distinguished the 30 codons that likely make up the peptide-binding region (PBR) [49], which is expected to be under strong positive selection due to its role in antigen binding specificity [41]. We conducted separate molecular sequence analyses on the PBR and non-PBR regions using MEGA 4.0 and the Jukes-Cantor model of sequence evolution [50]. For all analyses, we used 1,000 permutations where necessary and assumed the Kimura 2P model of evolution.

We used Arlequin 3.1 [51] to conduct analyses of molecular variance [52], and to quantify pairwise F_st_ values across our four populations (pre-epidemic eastern, post-epidemic eastern, pre-control western, post-control western) via Mantel tests. For the genetic structure analyses, we predicted that the MG epidemic would result in significant genetic structure between pre- and post-epidemic eastern populations whereas we would not detect genetic structure between control populations sampled over the same time period (pre-control versus post-control western).

## Results

### (a) Experimental challenge

**Figure 1:**
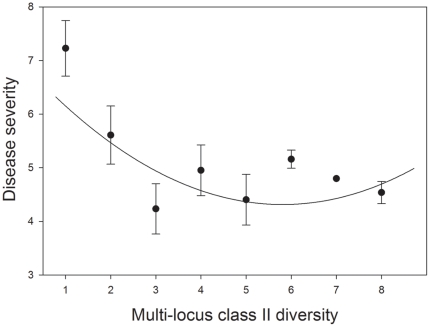
Multi-locus class II major histocompatibility complex (MHC) diversity and house finch disease resistance. MHC class II diversity as measured by single strand nuclear polymorphism predicts the severity of house finch (*Carpodacus mexicanus*) conjunctivitis in response to experimental infection with *Mycoplasma gallisepticum* (MG). All bars indicate one standard error around the mean.

House finches with intermediate to high multi-locus class II MHC diversity, as measured by the number of segregating SSCP bands, showed the lowest disease severity in response to experimental infection with MG (Figure 1; *n* = 39, *r^2^* = 0.34; effect tests: *SSCP diversity t* = −3.18, *p*<0.001; SSCP diversity*SSCP diversity t = 2.59; *p* = 0.01). The model including MHC diversity as a second-order relationship was a significantly better fit when compared to the model including MHC diversity as a first-order relationship alone (*D* = 3.71; df = 1; *p* = 0.05). Although not statistically significant, higher microsatellite heterozygosity tended to be associated with lower disease severity (*microsatellite heterozygosity t* = −1.88; *p* = 0.069), consistent with prior results in this system [47].

### (b) MHC and microsatellite diversity

**Figure 2:**
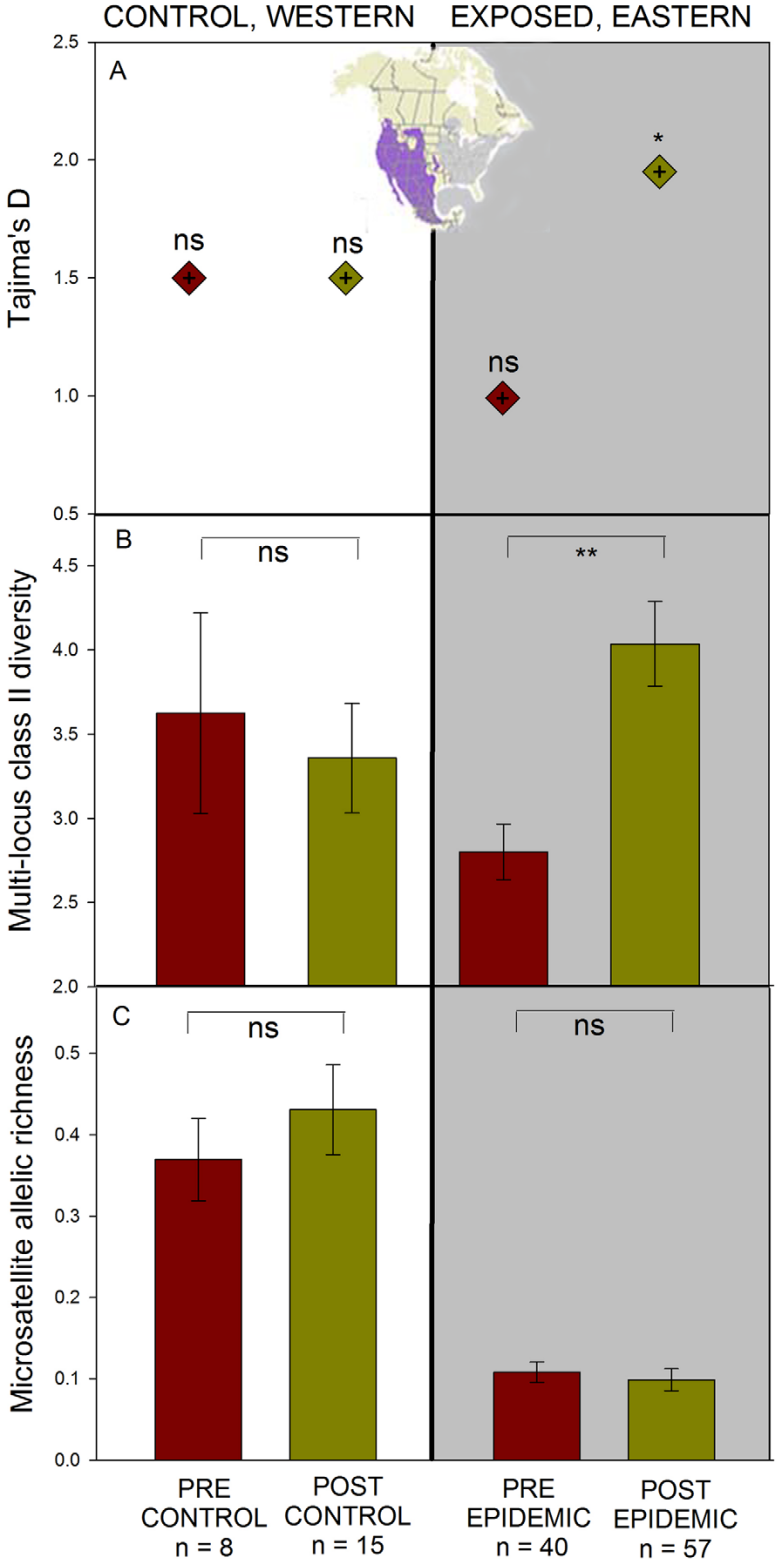
Epidemic associated changes in functional and neutral genetic diversity in house finches (*Carpodacus mexicanus*). Red indicates “pre” and yellow “post” values for control, western (left) and exposed, eastern (right) house finch populations across equivalent time periods. A) Tajima's D calculated from a portion of exon 2 of the class II Major Histocompability Complex (MHC) B) individual-level diversity amplified from multi-locus MHC class II, and C) microsatellite allelic diversity. Inset map shows the native (left) and introduced (right) range of the house finch. All bars indicate one standard error (s.e.m.) around the mean (*p<0.05; **p<0.01).

Prior to the emergence of MG, eastern house finch populations had significantly lower individual-level class II MHC diversity than native western house finches (Figure 2*B*; *F_1,60_* = 4.46; *p* = 0.039). These results reflect a ∼23% loss of MHC diversity as a result of the introduction. Individual-level MHC diversity increased significantly following the MG epidemic in eastern house finches but did not change over an equivalent time period in control, western populations (full model *F_3,114_* = 4.94; *p* = 0.0029; effect test population*time: *F_1,117_* = 3.88; *p* = 0.05, *population*, *time* effect tests *F_1,118_*<0.84; *p*>0.21). As a result, eastern and western house finches did not differ in individual-level MHC variability following the MG epidemic (Figure 2*B*), but still differed in microsatellite diversity to an equal extent (Figure 2*C* *; see results below*). We also tested whether our MHC results held true when we limited the analysis to the Tompkins Co., NY population, for which we had a large number of both pre- and post-epidemic samples (*n* = 72). We detected a significant increase in MHC diversity consistent with our broader meta-population patterns within Tompkins Co., NY (Pre-MG: 3.06±0.45 variants: post MG: 4.04±0.23 variants; *t_41_* = −2.66; *p* = 0.01), suggesting that the detected increase in MHC diversity across the eastern range is not biased by the inclusion of multiple populations.

As detected previously (33), western native house finches had significantly higher microsatellite allelic richness than eastern, introduced populations at both time periods examined (Figure 2*C*; full model *r^2^* = 0.68, *F_3,28_* = 20.2; *p*<0.0001; population effect test *F_1,28_* = 59.2, *p*<0.0001). Pre- and post-epidemic or control house finch populations did not differ significantly in allelic richness (*time*, population*time effect tests: *F_1,28_*<0.83; *p*>0.37). Mean heterozygosity across loci was statistically equivalent for all populations and over time (full model: *r^2^* = 0.04, *F_3,28_* = 0.46; *p* = 0.71; effect tests for *population*, *time*, and population*time F_1,28_<0.73, *p*>0.39). However, consistent with prior work (32), mean heterozygosity at both time periods was lower for introduced, eastern populations (pre-epidemic: 0.71±0.06; post-epidemic: 0.75±0.06) than for western, native populations (pre-control: 0.75±0.06; post-control: 0.81±0.06).

### (c) MHC sequenced-based analyses

Sequence-based indices tracked the SSCP diversity patterns (Table 3): Tajima's D, a measure of the extent to which gene sequences do not fit a null model of equilibrium between mutation and genetic drift [53], significantly increased in eastern populations following the MG epidemic, but did not change in western house finch populations across an equivalent time period (Figure 2*A*). Ratios of non-synonymous to synonymous substitutions (d_n_/d_s_), with high values (>1) signifying positive molecular selection [but see 54]–[55], increased significantly in eastern populations across the putative peptide-binding region following the MG epidemic but did not change in western populations. However, these changes were due to decreases in d_s_ values, rendering the result difficult to interpret.

**Table 3:** MHC class II sequence diversity for MG-affected eastern populations and unaffected control populations across equivalent time periods.

Population	N	V	Gene diversity	Θ(obs)	Taj D	d_n_: PBR	d_s_: *PBR*	d_n_/d_s_: PBR
						*non-PBR*	*non-PBR*	*non-PBR*
**Pre-MG**	73	33	0.95±0.01	15.6	0.99	0.26±0.06	0.085±0.03	3.06
						*0.07±0.03*	*0.079±0.05*	*0.86*
**Post-MG**	94	40	0.96±0.008	13.9	1.95	0.24±0.06	0.045±0.02	5.33
						*0.08±0.04*	*0.103±0.06*	*0.78*
**Pre-Control**	18	17	0.99±0.02	14.8	1.50	0.23±0.07	0.072±0.03	3.19
						*0.07±0.03*	*0.044±0.04*	*1.59*
**Post-Control**	25	18	0.97±0.02	15.4	1.50	0.26±0.09	0.077±0.03	3.71
						*0.10±0.04*	*0.121±0.05*	*0.83*

N = no. unique gene copies; V = no. unique sequence variants; Taj D = Tajima's D; PBR = peptide binding region (30 codons out of 55 total); MG = *Mycoplasma gallisepticum*.

### (d) MHC variant frequency

Western, native populations of house finches harboured, on average, 1.5 times the number of rare MHC variants (i.e. variants amplified from only a single individual in each population) than that of eastern, introduced house finches both prior to and following the epidemic: in our pre-epidemic subsample which standardized for sample size across populations (*n* = 8 for both), western house finches harboured 15 rare variants, 1.53 times that of eastern populations (9.8 rare variants). These differences were similar post-epidemic (*n* = 14 for both), where western populations (15 variants) harboured 1.47 times the number of rare variants as eastern populations. Overall, these results reflect a ∼35% loss of rare MHC variants as a result of the introduction bottleneck.

### (e) Genetic Structure: MHC and microsatellites

The analysis of molecular variance revealed that the majority (98.6%) of molecular variance for our MHC sequences was detected within populations (eastern pre-epidemic, eastern post-epidemic, western pre-control, and western post-control; *V_c_* = 11.9; *p* = 0.000). Differences between pre- and post-control western populations accounted for only 0.31% of detected variation (*V_a_* = 0.12; *p* = 1.00) while differences between pre- and post-epidemic eastern populations explained 1.35% of the total molecular variance (*V_b_* = 0.16; *p* = 0.16).

Consistent with our predictions, pairwise F_st_ values for the MHC exon were statistically significant between pre- and post-epidemic eastern populations but not between pre- and post-control western populations (Table 4). Statistically significant pairwise F_st_ values were detected between all population pairs except the pre- and post- western control populations at the eight examined microsatellite loci.

**Table 4:** Genetic structure (pairwise F_st_ values) for the MHC and microsatellite loci examined (microsatellite results shown in parentheses).

	Pre-Control	Post-Control	Pre-MG
**Pre-Control**	--------	--------	--------
**Post-Control**	0.0044 (0.023)	--------	--------
**Pre-MG**	−0.017 (0.031*)	0.041* (0.046*)	--------
**Post-MG**	−0.008 (0.034*)	0.023* (0.041*)	0.013* (0.003*)

*p≤0.05; MG = *Mycoplasma gallisepticum*.

## Discussion

We detected molecular and diversity-based signatures of balancing selection on house finch MHC class II loci that together indicate that a pathogen epidemic can exert significant balancing selection on host MHC variation in only 5–7 host generations. Although the introduction bottleneck of eastern house finches resulted in significant reductions of multi-locus diversity, individual-level MHC diversity returned to pre-bottleneck, native levels following the *Mycoplasma* epidemic while neutral microsatellite diversity remained equivalently compromised (Figure 2). Changes in individual-level MHC diversity were not observed over an equivalent time period in our control, western house finch populations which remained unexposed to the *M. gallisepticum* epidemic during our sampling period. Second, in contrast to prior work [56], we detected a significant increase in Tajima's D in eastern, exposed populations following the MG epidemic while no similar changes were documented in unexposed, native populations. High positive values of Tajima's D generally reflect balancing selection and/or a recent population bottleneck [53]. Although we cannot eliminate the possibility that higher Tajima's D values in eastern, exposed populations reflect a pathogen-induced population bottleneck [57], levels of microsatellite allelic diversity which are highly sensitive to population demographic changes did not change over the same time period (Figure 2*C*). Furthermore, the consistency of balancing selection signatures that we detected using disparate measures (diversity- and sequence-based) reduces the likelihood that bias in any one component of our study produced the detected results.

Balancing selection likely resulted in this system because the emergence of MG selected for house finches capable of responding to as many foreign antigens as possible (i.e. those individuals with higher variant diversity). Harboring a larger suite of receptor-variants increases the probability that at least one of those variants is capable of initiating a T-cell response in response to one or more *M. gallisepticum* antigens [2]. Our experimental results support this mechanism (Figure 1): house finches harboring intermediate to high numbers of MHC variants showed significantly lower pathology upon challenge with equal doses of MG. Equivalent experimental results have been detected in three-spined sticklebacks (*Gasterosteus aculeatus*), where low MHC variant diversity is associated with increased susceptibility to infection with both tapeworms and microsporidians [48]. Although balancing selection at MHC loci has been demonstrated to result from resistance to a range of potential pathogens [32], [58], MHC diversity is equally likely to benefit resistance to single pathogens given the large number of immuno-antigens produced by any given infection. For example, up to ten immunoantigens were detected in wild birds infected with *Borrelia spp.* bacteria [59]. Furthermore, *M. gallisepticum* is known to rapidly alter expression of its surface antigens during infection in poultry [60]. If similar mechanisms occur in house finches, the presence of multiple immuno-antigens within an individual house finch would provide a direct benefit for disease resistance.

We were unable to distinguish between the two mechanisms of balancing selection (heterozygote advantage versus frequency-dependent selection) that may have acted in this system due to the unknown locus identity for our amplified variants. Identifying single-locus primers in passerines such as the house finch may be possible with the recent characterization of the zebra finch MHC [21], and single-locus genotyping will be critical in order to identify the mechanisms of MG-mediated selection acting on house finch class II MHC [61]. Although the primary form of selection remains unknown, our analysis of variant frequency indicates that MHC diversity increased via selection on existing variation rather than the generation of novel diversity via immigration or mutation. The introduction bottleneck reduced the number of rare MHC variants in eastern house finch populations by approximately 35% relative to western, control populations. However, the frequency of rare MHC variants did not increase following the MG epidemic as would be expected if immigration or mutation had introduced novel MHC variation during that time period. Instead, population-level diversity of MHC remained low in eastern, introduced populations while average individual-level diversity increased as a result of the *Mycoplasma* epidemic (Figure 1*B*). The detected increase in individual-level MHC diversity therefore appears to have resulted from pathogen-mediated balancing selection on individuals with higher numbers of MHC variants.

Several alternative mechanisms that may also explain the detected patterns cannot be ruled out by the present study. Individual-level diversity at the examined MHC exon may not directly mediate house finch immune responses to MG antigens, but may instead be linked or correlated with an unmeasured gene(s) important for resistance. Given that the microsatellite loci distributed throughout the genome did not change in diversity following the epidemic, it is unlikely that MHC diversity in this study reflects a general measure of genetic diversity but rather a more specific response, either at the exon examined or closely-linked immune loci. Indeed, MHC regions are known to show high levels of linkage disequilibrium [62]. On the other hand, Hess et al. [55] demonstrated that house finch class II MHC is downregulated in response to experimental infection with MG. Although their findings confirm that class II MHC responds to MG infection and therefore may be important for infection response, the detected downregulation raises intriguing questions regarding the role of MHC class II variant diversity during MG infection.

The technique we employed for measuring individual-level, multi-locus MHC diversity—SSCP segregation — is appropriate for passerine class II MHC loci, where gene conversion has resulted in suites of highly similar loci whose alleles are often indistinguishable via standard PCR techniques [18]. Although our technique is a sampling approach that is unlikely to amplify all functionally-similar class II MHC variants present in the population, our results (Figure 1) indicate that house finches harbor a minimum of five class II loci. The finding of some individuals with only one or two variants (Figure 1) raises the possibility that house finches vary in their overall number of class II loci and/or exhibit high levels of allele sharing across loci, and both sources of variation likely contribute to functional MHC diversity. This result is not surprising: evidence for variable numbers of class II loci among individuals within a species and/or allele sharing across loci have been documented in a suite of vertebrate systems, including fish, mammals, and birds [e.g. 26]–[29]. The extent to which individuals vary in their number of class II loci has remained difficult to quantify in the absence of cost-effective genomic techniques, but recent advances in pyrosequencing [63] suggest this information will be accessible in the near future for a suite of non-model systems.

The multi-locus technique employed here allowed us to sample a broad but not exhaustive set of functionally equivalent loci. However, the inability to pinpoint locus identity, and consequently, the inability to measure locus-specific heterozgosity, remains a strong drawback. Recently, Rakus et al. [64] linked SSCP banding patterns directly with locus-level heterozygosity in carp, providing direct evidence in one system that the SSCP sampling approach samples meaningful diversity. Although we were unable to conduct an equivalent analysis, we used a conservative methodological approach in this study in order to ensure that our SSCP technique captured meaningful variation. First, we cloned the exon of interest from the PCR products of 16 individuals for which we had also segregated variants via SSCP and found identical variant numbers regardless of the technique used. Second, we only included variants in our final sequence analysis that we were able to amplify and sequence in duplicate, eliminating potential errors due to contamination or Taq polymerase during cloning. Third, we validated our SSCP banding patterns by running single variant clones alongside multi-variant amplifications, confirming that SSCP banding patterns corresponded to individual sequence variants. The results of these checks indicate that our SSCP technique was repeatable and robust.

The use of museum DNA for the majority of our pre-epidemic samples raises an alternative hypothesis that detected increases in MHC diversity reflect increasing amplification likelihood of rare variants in higher quality DNA. We used two types of controls to eliminate this possibility. First, if MHC diversity increased over time due to amplification likelihood alone, we would expect to see this pattern in our control population. In contrast, pre-control samples from western house finch populations show equal levels of diversity to post-control samples despite equal potential for degradation of museum DNA in this population. Second, we included tissue samples wherever possible in our pre-epidemic samples, and used direct side-by-side comparisons of tissue and toe-pad samples for six individuals to demonstrate comparable amplifiability of freshly collected blood and 10–20 year old museum specimens (see *methods* for details). Finally, although we pool a number of geographic areas in our pre- and post-epidemic samples, our restricted analysis of Tompkins Co., NY alone indicates that it is unlikely that population genetic structure, a potential selective mechanism underlying MHC polymorphism [7]–[9], [65], influenced our results.

The extent to which population bottlenecks compromise functional genetic variability for pathogen resistance in threatened vertebrate populations continues to be of strong concern [66]–[67] particularly because in some cases, the loss of diversity at functional loci such as MHC class II is of significantly greater magnitude than losses in neutral diversity [68]. Somewhat paradoxically, many wild vertebrate populations that experienced known demographic bottlenecks show little to no loss of MHC diversity [15] or regain MHC diversity in only 10–20 generations, as was detected in the endangered San Nicolas Island fox (*Urocyon littoralis dickeyi*) [69]. These systems suggest that strong pathogen-mediated balancing selection and/or mate choice [70] may be sufficient to maintain or recover MHC variability in host populations following considerable demographic losses. Here we documented that the introduction bottleneck of eastern house finches compromised levels of individual-level MHC diversity by ∼23%, equivalent to the ∼18% reduction previously detected for microsatellite allelic richness [39], but intriguingly, these differences disappeared following the *Mycoplasma* epidemic (Figure 1*B*). A common garden study of contemporary (2007–08) eastern and western house finches [71] showed no differences in the extent of individual-level MHC class II diversity, consistent with the results detected here. The strong selection that MG placed on eastern house finches, with selection coefficients as high as 0.6 during the early part of the epidemic [36], may explain the rapid changes in diversity seen here if more heterozygous finches and/or finches harboring a larger number of class II variants were more likely to survive the epidemic. Indeed, the potential for rapid evolution in this system was recently confirmed by Bonneaud et al. (2011) who found evidence for changes in house finch gene expression in response to MG infection over the course of only 12 years of population-level exposure to MG epidemics in the eastern United States [72].

In conclusion, the unusually high fitness consequences of the house finch-MG epidemic, in combination with the discrete time period and geographic range over which the epidemic occurred, make it a particularly valuable system for directly linking pathogen-mediated selection with changes in MHC class II genes in a natural vertebrate population. In so doing, the house finch system provides direct evidence for Haldane's long-standing yet largely untested hypothesis that pathogens contribute to the maintenance of the tremendous levels of genetic variation detected in natural populations of vertebrates [1]. As techniques continue to rapidly improve for isolating and amplifying museum quality nuclear DNA [73], historical studies of host-pathogen systems will shed further light on the extent to which pathogen-mediated selection can act on immunogenetic loci in natural populations.
